# Immune Modulation of Cardiac Repair and Regeneration: The Art of Mending Broken Hearts

**DOI:** 10.3389/fcvm.2016.00040

**Published:** 2016-10-14

**Authors:** Ivana Zlatanova, Cristina Pinto, Jean-Sébastien Silvestre

**Affiliations:** ^1^UMRS-970, Paris Centre de Recherche Cardiovasculaire, Institut National de la Santé et de la Recherche Médicale (INSERM), Sorbonne Paris Cité, Université Paris Descartes, Paris, France

**Keywords:** cardiovascular diseases, myocardial infarction, inflammation, myocytes, cardiac, regeneration, remodeling pathways

## Abstract

The accumulation of immune cells is among the earliest responses that manifest in the cardiac tissue after injury. Both innate and adaptive immunity coordinate distinct and mutually non-exclusive events governing cardiac repair, including elimination of the cellular debris, compensatory growth of the remaining cardiac tissue, activation of resident or circulating precursor cells, quantitative and qualitative modifications of the vascular network, and formation of a fibrotic scar. The present review summarizes the mounting evidence suggesting that the inflammatory response also guides the regenerative process following cardiac damage. In particular, recent literature has reinforced the central role of monocytes/macrophages in poising the refreshment of cardiomyocytes in myocardial infarction- or apical resection-induced cardiac insult. Macrophages dictate cardiac myocyte renewal through stimulation of preexisting cardiomyocyte proliferation and/or neovascularization. Nevertheless, substantial efforts are required to identify the nature of these macrophage-derived factors as well as the molecular mechanisms engendered by the distinct subsets of macrophages pertaining in the cardiac tissue. Among the growing inflammatory intermediaries that have been recognized as essential player in heart regeneration, we will focus on the role of interleukin (IL)-6 and IL-13. Finally, it is likely that within the mayhem of the injured cardiac tissue, additional types of inflammatory cells, such as neutrophils, will enter the dance to ignite and refresh the broken heart. However, the protective and detrimental inflammatory pathways have been mainly deciphered in animal models. Future research should be focused on understanding the cellular effectors and molecular signals regulating inflammation in human heart to pave the way for the development of factual therapies targeting the inflammatory compartment in cardiac diseases.

Shortly after birth, mammalian cardiac myocytes escape the cellular cycle, and heart growth is mainly mediated by hypertrophy of preexisting cardiomyocytes. However, over the last decade, the classical dogma stamping that adult mammalian heart displays no cell renewal/replication capability has been challenged. Indeed, recent studies unravel that cardio-myogenesis, i.e., the formation of new cardiomyocytes, also occurs during adult life, including in human (1, 2). However, the intrinsic capacity of the adult mammalian heart to regenerate after injury, such as myocardial infarction (MI), is derisory to swap the loss of functional myocardium. In this view, it has been valued that after MI, a patient loses on average around 1 billion cardiomyocytes, a massive amount that the cardiac tissue cannot substitute by itself (3). As cardiac damage typically guides fibrotic scar formation and contractile dysfunction, cardiomyocyte loss after injury, aside from the failure of the human heart to regenerate, is a fundamental cause of heart failure and death worldwide. In addition, it is likely that a variety of issues, including age and cardiovascular risk factors, also fuel the deleterious micro- and macro-environments that dampen bona fide cardio-myogenesis as well as efficient tissue repair in patients with cardiac diseases.

Precisely orchestrated process of cardiac regeneration reestablishes tissue organization through a coordinated sequence of cellular proliferation, differentiation, dedifferentiation, and morphogenic redisposition (4, 5). Complete understanding of how cardiac tissue can regenerate could pave the way to factual therapeutic strategies of regenerative medicine for cardiac diseases. Multiple different approaches have been initiated to promote cardiomyocyte regeneration/proliferation in experimental models of heart injury, including transplantation of non-cardiac/cardiac somatic stem cells, injection of cultured cardiovascular progenitor or cardiomyocytes, direct *in vivo* reprograming of differentiated cells into cardiomyocytes, stimulation of dedifferentiation/proliferation of preexisting cardiomyocytes, and activation of endogenous cardiac progenitor cell populations. These therapeutic approaches are categorized as either cell-based or cell-free, and some of them are currently being tested for their cardiac regenerative ability, safety, and feasibility of clinical application. However, the mixed results obtained in a variety of clinical trials performed to date, especially for cardiogenic stem cell-based therapy, do not conclusively prove the reality of cardiac myocyte refreshment and the efficiency of these strategies, so far.

## Inflammation and Cardiac Repair

Mammals respond to heart damage, such as MI, through distinct and mutually non-exclusive events, including elimination of the cellular debris, compensatory growth of the remaining myocardium, activation of resident or circulating precursor cells, quantitative and qualitative modifications of the vascular network, and formation of a fibrotic scar.

### Inflammation and Mechanisms of Cardiac Repair

**Figure 1:**
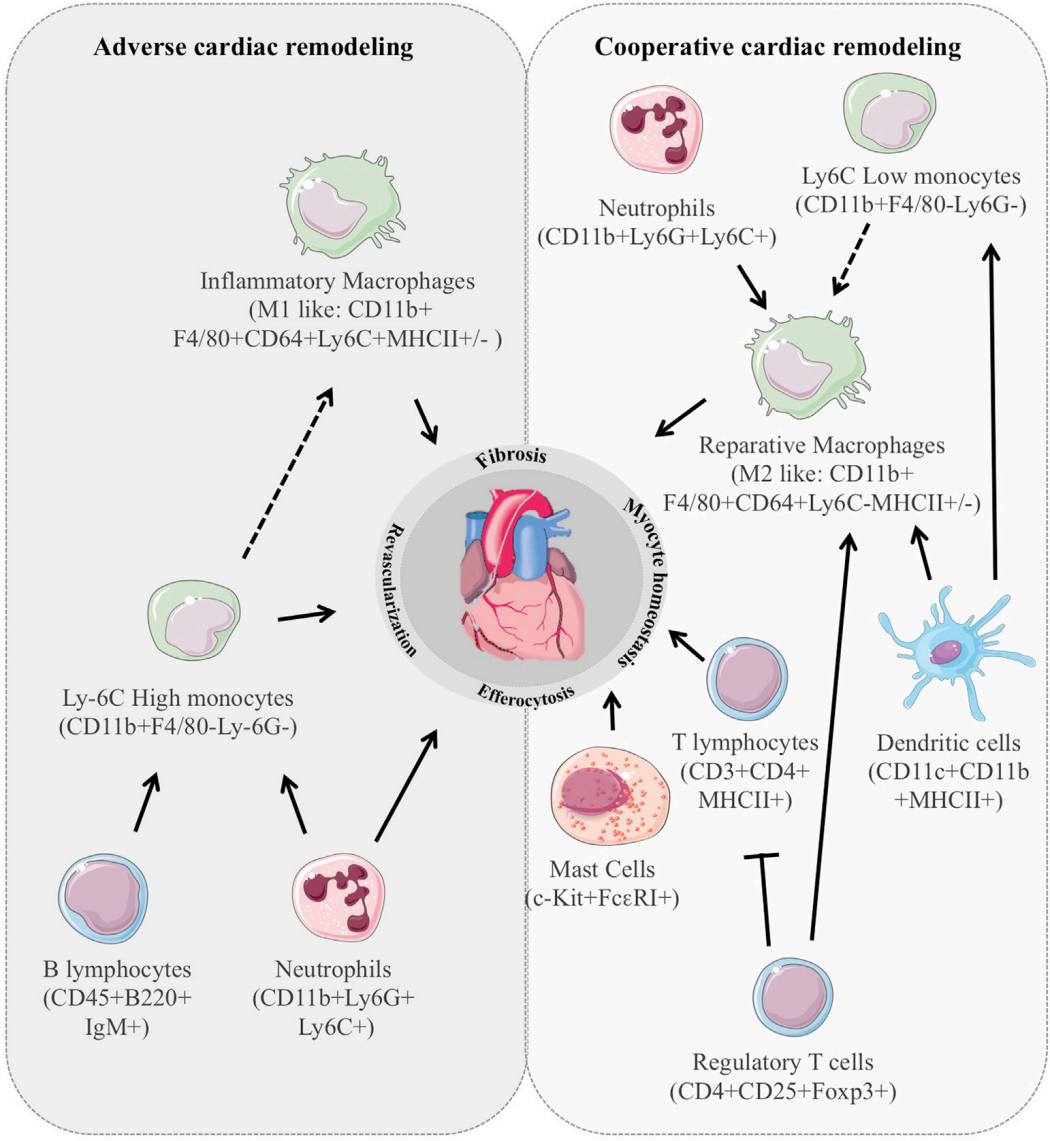
**Immune cell stimulation is among the earliest responses detectable in the injured cardiac tissue and plays an instrumental role in the coordination of multiple processes governing cardiac remodeling**. In animal models, the number, type, and activation state of the different subclasses of inflammatory cells dictate their impact on cardiac repair leading to either positive or deleterious cardiac remodeling.

Remarkably, immune cell stimulation is among the earliest responses detectable at the infarcted site after MI and plays an instrumental role in the coordination of multiple processes governing cardiac repair namely survival of resident cells, removal of dying cells fibrosis, infarct size, and revascularization (Figure 1). Indeed, the early inflammatory phase depicted by an intense sterile inflammation and immune cell infiltration serve to digest and clear damaged cells and extracellular matrix tissue and is followed by a reparative phase with resolution of inflammation, (myo)fibroblast proliferation, scar formation, and neovascularization over the next several days (6, 7). An appropriate equilibrium between these 2 phases is mandatory for optimal repair. In particular, suitable, timely confinement and resolution of inflammation are key elements of the quality of cardiac healing. Consistent with this, deregulation of molecular mechanisms involved in inflammation resolution, such as deficiency for the decoy receptor D6, exacerbates inflammatory cell infiltration, and adverse ventricular remodeling (8). Similarly, improper clearance of dying cells by activated neighboring phagocytes delays and hampers the reparative phase worsening cardiac healing (9, 10). Inflammation also directly shapes cardiomyocyte hypertrophy. In particular, neutrophils and T lymphocytes are involved in the modulation of cardiomyocytes size in experimental model of cardiac hypertrophy (11, 12). Macrophages have been shown to release prohypertrophic factors (13) and antibody against IL-6 hampers cardiomyocyte hypertrophy in mice with MI (14). Finally, cardiomyocyte homeostasis determines the nature and intensity of the inflammatory reaction. Release of the cytokine oncostatin M by infiltrating neutrophils and macrophages prompts a positive feedback loop in which oncostatin M galvanizes cardiomyocytes to produce REG3β that in turn attracts additional macrophages to the damaged heart (15). Mechanical strain through mitogen-activated protein kinase dependent pathways has also been shown to expand the macrophage population in the remote non-infarcted myocardium of the failing heart (16).

Overall, the net effect of the various actors of the inflammatory component is determined by their number, activation, and differentiation states. In addition, the local environment and the different stimulated pathways also balance the resultant influence of each inflammatory entity. For example, whereas interleukin (IL)-10 displays a strong and potentially deleterious antiangiogenic effect in ischemic tissue (17, 18), it also reduces reactive hypertrophy and myocardial collagen deposition leading to cardiac protection after MI (19).

### Myeloid Cells

The cellular components of innate immunity are myeloid cells, including monocytes, macrophages, mast cells, dendritic cells (DC), natural killer cells, as well as neutrophilic, basophilic, and eosinophilic granulocytes. Neutrophils are the first immune cell type to massively populate the infarcted myocardium in response to such factors as danger-associated molecular pattern, cytokines and chemokines, or endogenous lipid mediators. Neutrophils release proteolytic enzymes and contribute to the clearance of the wound from dead cells and matrix debris. Secretion of neutrophil gelatinase-associated lipocalin by cardiac neutrophils favors the occurrence of macrophages with reparative phenotype, thereby facilitating tissue healing (20). However, infiltrating neutrophils may also amplify the immune response (21) and exert direct cytotoxic actions on viable cardiomyocytes expanding ischemic injury (22, 23). Decreased number of CD209^+^ DC and CD11c^+^ DC in human-infarcted myocardial tissue correlates with increased macrophage infiltration, impaired reparative fibrosis, and the development of cardiac rupture after MI (24). Mice with DC ablation show deteriorated left ventricular function and remodeling and sustained expression of inflammatory cytokines, such IL1β, IL18, and tumor necrosis factor-α (25). Nevertheless, the specific role of each DC subtypes, such as conventional/classical DC or plasmacytoid DC, remains to be defined. In addition, multipotent progenitor cells identified as myeloid-derived suppressor cells (MDSC) are also known to infiltrate the infarcted heart, 24 h after acute MI (26). MDSC are mainly characterized as CD34^+^, Gr1^+^, or CD11b^+^ cells. Cytology and gene expression studies support MDSC heterogeneity and distinguishing MDSC from macrophages or neutrophils can be technically challenging. Functionally, they inhibited T cell proliferative responses, antibody production, and cytotoxic T lymphocyte induction and activity (27). In addition, Gr1^low^CD11b^+^ cells have been shown to improve vessel density and blood flow recovery in ischemic tissue suggesting that MDSC could also control vascular remodeling in the infarcted heart (28).

Other types of myeloid cells are also known to control cardiac repair through distinct mechanism. Mast cells deficiency has been shown to hamper postischemic cardiac function and reduced cardiomyocyte contractility caused by myofilament calcium desensitization through a tryptase dependent mechanism (29). Interestingly, the infarcted heart hosts a mast cell lineage that derives from the adipose tissue and does not arise from bone marrow progenitors (29, 30). One can then speculate that the adipose tissue constitutes an additional reservoir of myeloid progenitors that could play a specific role in cardiac diseases, especially during diabetes or obesity.

### Circulating Monocytes

After the prompt appearance of neutrophils, monocytes, and macrophages comprise the most abundant cells in the infarcted heart. Mobilization of monocytes from both bone marrow and splenic reservoir translates into two successive periods of monocyte infiltration in the infarcted myocardium (31, 32). The inflammatory Ly-6C^high^ monocyte subset is recruited during the first days after MI but vanishes thereafter when inflammation resolves in the tissue wound. Starting at approximately day 3 after MI, the infarct tissue also amasses Ly-6C^int/low^ monocytes. The inflammatory Ly-6C^high^ monocyte subset fosters vigorous inflammation and proteolysis, whereas Ly-6C^int/low^ monocytes seem to promote reparative activities (31, 32). In this line, inhibition of CCR2, the CCL2 chemokine receptor that governs Ly-6C^high^ monocyte mobilization from the bone marrow and recruitment to the cardiac tissue, attenuates infarct inflammation, and curbs post-MI left ventricular remodeling (33, 34).

### Monocyte-Derived Macrophages and Resident Macrophages

The majority of these infiltrated monocytes may exit the cardiac tissue through systemic or lymphatic circulation, whereas surviving monocytes may differentiate into different subgroups of macrophages with M1-like and/or M2-like activation mode associated with specific functions in cardiac homeostasis (35). However, it is noteworthy that this classification as M1 and M2 is based on *in vitro* activation by T-helper cell-type 1 (Th1) or Th2 cytokines only illustrating two extreme, opposing activation states. Hence, such nomenclature does not reflect the spectrum of functionally overlapping macrophage phenotypes in the cardiac milieu (36). Consequently, interpreting the published literature is difficult. More importantly, most of the results are obtained in experimental models, and the translation to the human immune system remains speculative. As a prototypic example, human blood monocytes are defined as CD14^+^CD16^−^ classical, CD14^+^CD16^+^ intermediate, and CD14^low^CD16^+^ non-classical monocytes. CD14^low^CD16^+^ human monocytes are likely the counter parts of Ly-6C^low^ mouse monocytes, whereas CD14^+^CD16^−^ human monocytes are that of Ly-6C^high^ mouse monocytes (37). To facilitate translation from mouse to humans, a unified nomenclature should be applied across tissues and species. In this line of reasoning, it has been proposed that monocytes and macrophages should first be defined on the basis of their progeny and second on the basis of their function, location, and/or phenotype (38).

To add on complexity, genetic fate mapping demonstrated that macrophages derived from CX3CR1^+^ embryonic progenitors persisted into adulthood (39). Nevertheless, the nature of those fetal precursors is still a matter of debate since conflicting results suggest that adult tissue-resident macrophages are all derived from yolk sac erythro-myeloid precursors or are progenies of classical hematopoietic stem cells with the exception of microglia and partially epidermal Langerhans cells (40, 41). These specific subclasses of resident macrophages of embryonic origin have also been shown to impact cardiac repair (42–45). However, after injury, these cells are replaced by monocyte-derived macrophages that are proinflammatory and lack reparative activities (42). Furthermore, embryo-derived cardiac macrophages show declining self-renewal with age and are progressively substituted by monocyte-derived macrophages, even in the absence of injury (39). Thus, more research is needed to determine the exact origin, number, and function of these adult cardiac tissue-resident macrophages as well as to assess their prominence in the human heart.

### Lymphoid Cells

The central cellular components of adaptive immunity are T- and B-cells that result from lymphoid progenitor cells in the bone marrow. CD4^+^ and CD3^+^ T lymphocytes promptly colonized the infarcted heart, whereas B lymphocytes peaked later one around day 5 after the onset of ischemia (46). Lymphocyte activation by myocardial auto-antigens, recognized either by their respective T- or B-cell receptor or, alternatively, by pattern recognition receptors, shapes the dysfunctional infarcted heart either through their ability to release fibrotic factors, such as transforming growth factor β and IL-13, or indirect interaction with innate immunity (47). In this view, absence of CD4^+^ T-cells improves the amount of Ly-6C^high^ monocytes, reduces neovascularization, and collagen deposition, 7 days after MI (48). CD4^+^ CD25^+^ Foxp3^+^ regulatory T cell depletion is associated with M1-like macrophage activation, characterized by decreased expression of inflammation-resolving and healing-promoting factors. Despite marked proangiogenic effects, regulatory T cell ablation results in aggravated cardiac inflammation and deteriorated cardiac function (49, 50). Regulatory T cell activation by superagonistic anti-CD28 monoclonal antibody administration leads to M2-like macrophage differentiation and improves healing as well as survival after MI (50). Similarly, mature B lymphocytes selectively produce Ccl7 and induce Ly-6C^high^ monocyte mobilization from the bone marrow and recruitment to the heart, leading to enhanced tissue injury and deterioration of myocardial function (46).

## Inflammation and Cardiac Regeneration

The immune system is integral to the initial development of an organism as well as the continuous replacement of differentiated cell types to sustain homeostasis. Unlike embryonic development, tissue regeneration is initiated by an injury and mounting evidences suggest that the inflammatory response to that insult also guides the regenerative process. Nevertheless, whether immune activation prompts tissue regeneration or scarring is defined by a multiplicity of factors, including age, species, and the accessibility of a stem or progenitor cell pool.

Interestingly, neonatal mouse heart displays a transient regenerative potential and constitutes a valuable model to decipher the interaction between inflammation and mammalian cardiac regeneration. Nevertheless, the genuine ability of neonatal mouse cardiac tissue to regenerate following apical resection or MI-induced injury has been rapidly questioned (51). Methodological differences, such as variations in surgical technique, amount of resected myocardium, methods of assessment of resected and regenerated myocardium, and strategies of evaluation of myocyte proliferation, are likely to account for these discrepancies (52). However, it is clear that the type of injury limits the amount of refreshed tissue. Notably, permanent left anterior descending coronary artery ligation-induced MI produces incomplete regeneration with a residual small infarct in neonatal mouse (53). Nevertheless, substantial number of studies clearly proves that hearts of 1-day-old neonatal mice can regenerate after partial surgical resection or MI, through cardiomyocyte proliferation with minimal hypertrophy or fibrosis (54). Interestingly, a recent study reports the case of a newborn child having a severe myocardial infarction due to coronary artery occlusion. Cardiac recovery was observed within weeks after the onset of ischemia, indicating that, similar to neonatal rodents, newborn humans might have the inherent capacity to repair myocardial damage (55).

Newly formed cardiomyocytes can arise from preexisting cardiomyocytes or from proliferation/differentiation of a stem cell population. Genetic fate mapping indicates that the majority of cardiomyocytes within the regenerated tissue originates from preexisting cardiomyocytes in neonate mice after partial surgical resection (54) or in aging mice (56). Conversely, after MI or pressure overload, stem cells or precursor cells, which do not originate from the bone marrow, are able to refresh injured cardiomyocytes at a significant rate in adult tissue (56).

### Monocytes/Macrophages and Cardiac Regeneration

The early stages of cardiac regeneration are accompanied by an acute inflammatory response depicted notably by a marked infiltration of monocytes as well as upregulation of several inflammatory markers, such as IL6, IL1β, and Ccl3 (54). Moreover, injury-induced cardiomyocyte proliferation is inhibited after immunosuppression with dexamethasone (57). Neonatal mouse vagotomy has been shown to impair myocyte proliferation and heart regeneration after injury. Interestingly, neonatal vagotomy downregulates genes, such as IL1β and Cxcl5, involved in the innate immune response and chemotaxis (58).

**Figure 2:**
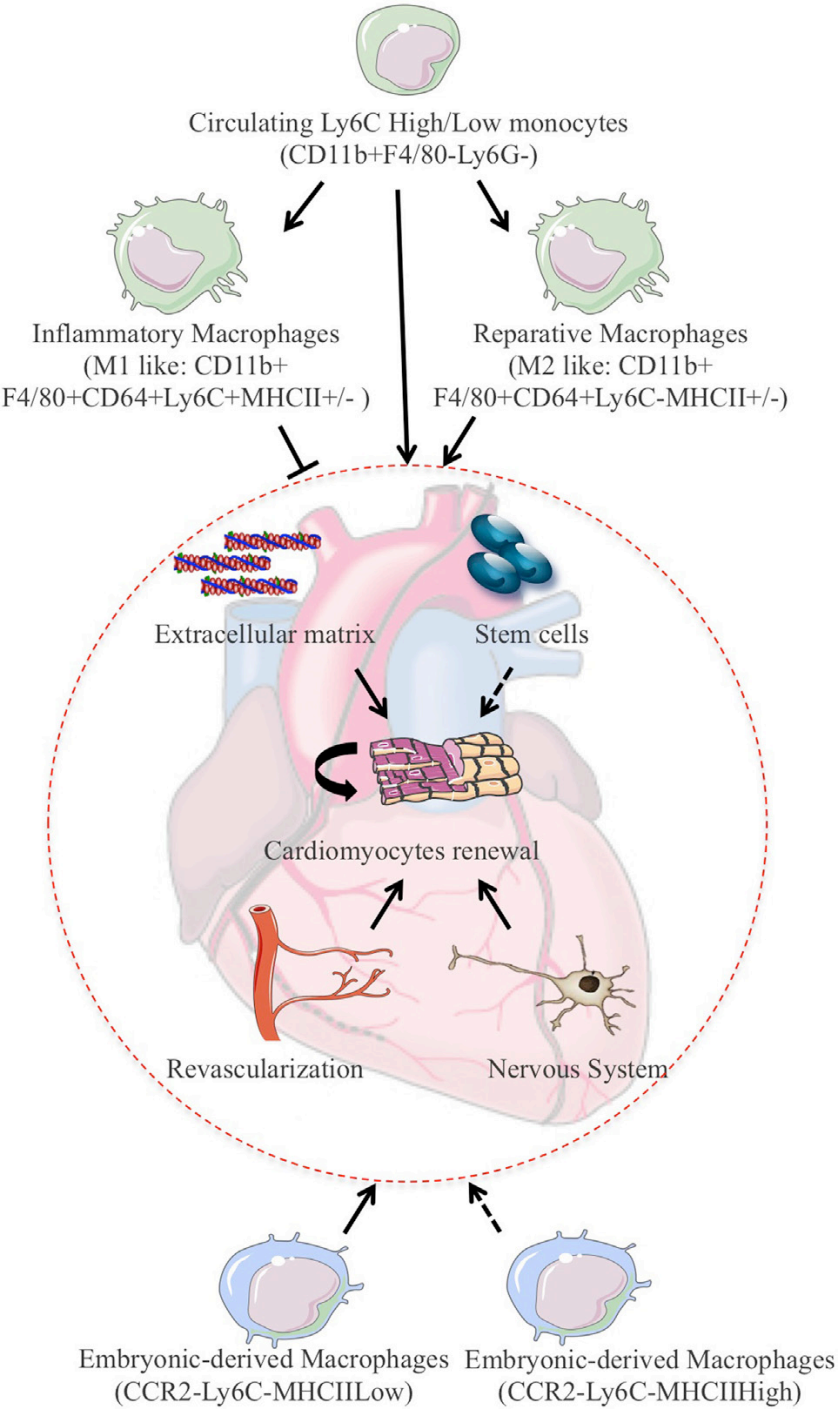
**In experimental models of MI, monocytes/macrophages fine-tune the balance between proliferation and repair and have been shown to promote cardiomyocytes refreshment after cardiac injury**. Cardiac regeneration can rely on the permissive environment for cellular proliferation induced by extracellular matrix deposition and activation of the vascular as well as the nervous compartment. One can also speculate that resident stem cells activation could also participate to cardiomyocytes refreshment in this setting. To add on complexity, resident macrophages of embryonic origin have drawn significant attention due to their counteracting roles on injury-induced inflammatory response and subsequent capacity for cardiac repair and regeneration.

Monocytes and macrophages seem to play a major role in cardiac refreshment in this experimental setting (Figure 2). Indeed, neonates depleted of macrophages are unable to regenerate myocardium and form fibrotic scars, resulting in reduced cardiac function and new vessel formation (59). Clodronate liposome-mediated depletion of monocytes/macrophages does not modulate cardiomyocyte proliferation in neonatal heart after MI but rather enhances cardiac regeneration *via* control of angiogenesis (59). Nevertheless, such approach is non-selective and drains all monocyte and macrophage subsets. As the different subsets of cardiac macrophages have distinct functional roles, it is likely that macrophage depletion prevents other reparative functions. In this line of reasoning, resident neonatal heart macrophages, not recruited monocytes, have been shown to mediate neonatal heart regeneration (42). The neonatal heart contained one embryo-derived macrophages (MHC-II^low^CCR2^−^) and one monocyte-derived macrophages (MHC-II^low^CCR2^+^) subset. In an *in vivo* mouse model of cardiomyocyte ablation that induces cardiomyocyte cell death without systemic inflammation and cardiac fibrosis, the neonatal heart selectively expands the number of MHC-II^low^CCR2^−^ macrophages and does not recruit additional CCR2^+^ monocytes. In contrast, the injured adult heart selectively recruits monocytes and MHC-II^high^CCR2^+^ monocyte-derived macrophages. Of interest, only CCR2^−^ neonatal macrophage conditioned media is sufficient to promote neonatal rat cardiomyocyte proliferation *in vitro* (42).

Hence, neonatal heart undergoes revascularization and cardiomyocyte proliferation after injury, whereas the injured adult heart exhibits minimal increases in vessels density and negligible cardiomyocyte proliferation (42, 59). In response to injury, the neonatal heart enlarges the number of resident CCR2^−^ reparative cardiac macrophages, whereas the adult heart enrolls CCR2^+^ proinflammatory monocytes and monocyte-derived macrophages. The presence of an embryonic pool of macrophages could explain the superior regeneration capacity of the neonatal heart and could promote regeneration versus repair. Depending on the inflammatory milieu, injury can then result either in complete tissue regeneration or in its deterioration and fibrosis. Consistent with this, in acutely damaged skeletal muscle, sequential interactions between multipotent mesenchymal progenitors and infiltrating inflammatory cells determine the outcome of the reparative process. In particular, the disruption of the precisely timed progression from tumor necrosis factor-rich to transforming growth factor-β-rich environment favors fibrotic degeneration of the muscle during chronic injury (60). In this view, immunophenotyping and gene expression profiling of cardiac macrophages from regenerating (day 1) and non-regenerating hearts (day 14) indicate that regenerative macrophages have a unique M2-like polarization phenotype and secrete numerous soluble factors that may facilitate the formation of new myocardium. Macrophage-derived factors dictate cell fate decisions and are involved in the regeneration of other organs, such as the kidney and the liver (61). Likewise, myogenic proliferation following toxic injury depends on monocyte/macrophage in skeletal muscle (62). Regeneration of experimentally induced demyelination is restored in old mice exposed to blood-derived monocytes from young parabiotic mice (63). Altogether, these studies suggest that monocytes/macrophages play critical roles in tissue regeneration in mammals.

#### Additional Types of Inflammatory Cells

Nevertheless, it is unlikely that only macrophages preside cardiac regeneration. One can speculate that additional actor of the immune system synchronizes the regenerative response in the mouse heart. In this line of reasoning, cardiomyocyte proliferation after apical resection or artificial inflammation induced by intra-myocardial injection of zymosan A is also associated with Ly-6G inflammatory neutrophils infiltration (57). Neutrophil numbers have also been shown to peak at day 1 following MI in neonate cardiac tissue (59). Similarly, the specific lymphocyte-dependent response has not been investigated in the cardiac tissue in this experimental model. However, in a model of skeletal muscle injury, a definite population of regulatory T cells has been shown to accumulate and switches from a proinflammatory to a pro-regenerative state. These muscle regulatory T cells express the growth factor amphiregulin, which acts directly on muscle satellite cells *in vitro* and improves muscle repair *in vivo* (64).

#### Inflammatory Mediators of Cardiac Regeneration

The ability of the different partners of cardiac inflammatory reaction to release inflammatory mediators is expected to coordinate cardiac refreshment. Among these inflammatory intermediaries, IL-6 emerges as an essential player in heart regeneration. Interestingly, both neutrophils and monocytes/macrophages are a rich source of IL-6 and its derivatives. IL-6 acts through activation of signal transducer and activator of transcription 3 (STAT3) downstream signaling. Cell-specific ablation of STAT3 in cardiomyocytes impairs their proliferative response after apical resection in neonatal mice (57). Interestingly, cardiomyocyte STAT3 activation is also required for injury-induced cardiomyocyte proliferation and heart regeneration in adult zebrafish (65).

Using RNA sequencing, IL13 has also been identified as a new regulator of cardiomyocyte cell cycle entry (66). IL-13 is a cytokine secreted by many cell types, but especially Th2 cells. IL4Ra/IL13Ra1 heterodimer mediates cardiomyocyte response to IL13 by the STAT3/periostin pathway suggesting that IL13/STAT3 could also be an initiating factor in mouse cardiac regeneration (66). Similarly, IL4/IL13 signaling is required for regeneration of skeletal muscle after injury. Muscle damage results in rapid recruitment of eosinophils, which secrete IL-4 to activate the regenerative actions of muscle resident fibro/adipocyte progenitors (67). Interestingly, IL4 increases the relative density of tissue macrophages in inflamed tissue without any substantial effect on the recruitment of circulating monocytes (68). Hence, IL4/IL13 regenerative effects may depend on a direct impact on cardiomyocytes but also on their ability to trigger the proliferation of resident CCR2^−^ reparative cardiac macrophages.

## Conclusion

Mounting evidences propose that monocyte- and embryonic-derived macrophages balance both regeneration and repair processes in cardiac tissue. However, the relevance of the data, obtained from the neonatal rodent heart, to the human adult heart is largely undetermined and the translation and repercussions of these experimental-based findings should be carefully evaluated. Nevertheless, although the adult myocardium undoubtedly displays a lower ability for cardiac repair and refreshment compared with the neonatal myocardium, it is likely that some aspects of repair or regeneration activities continue to exist. Hence, a deep comprehension of the molecular and cellular mechanisms governing the regenerative effect of distinct subset of macrophages is mandatory. Furthermore, a clear understanding of human macrophage plasticity and function could guide the development of specific macrophage-based therapies in patients with cardiac diseases. Finally, the putative involvement of other actors of the inflammatory reaction remains to be deeply appreciated, but additional types of inflammatory cells will likely enter the dance to ignite and refresh the broken heart.

## Author Contributions

IZ, CP, and J-SS perform literature search and writing. All authors approved the manuscript.

## Conflict of Interest Statement

The authors declare that the research was conducted in the absence of any commercial or financial relationships that could be construed as a potential conflict of interest.
